# Very large thermal rectification in bulk composites consisting partly of icosahedral quasicrystals

**DOI:** 10.1088/1468-6996/15/6/064801

**Published:** 2014-11-25

**Authors:** Tsunehiro Takeuchi

**Affiliations:** 1 Toyota Technological Institute, Nagoya 468-8511, Japan; 2 EcoTopia Science Institute, Nagoya University, Nagoya 464-8603, Japan; 3 PRESTO, JST, Tokyo 102-0076, Japan

**Keywords:** quasicrystal, thermal diode, electron thermal conductivity

## Abstract

The bulk thermal rectifiers usable at a high temperature above 300 K were developed by making full use of the unusual electron thermal conductivity of icosahedral quasicrystals. The unusual electron thermal conductivity was caused by a synergy effect of quasiperiodicity and by a narrow pseudogap at the Fermi level. The rectification ratio, defined by TRR = 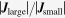, reached vary large values exceeding 2.0. This significant thermal rectification would lead to new practical applications for the heat management.

## 1. Introduction

Thermal rectifiers [1] have attracted considerable interest because of their ability to manage heat, a large fraction of which is typically lost into the environment. After the first report of thermal rectification [2], several different mechanisms leading to the thermal rectification were reported: (a) a metal-insulator junction [2], (b) thermal wrapping [3–7], (c) thermal strain at the interface [8], (c) the thermal potential barrier [9], (d) inhomogeneous mass loading [10] and (e) composite of bulk materials possessing different temperature dependences of thermal conductivity [11–15]. Among these thermal rectifiers, (e) the composite of two bulk materials of different temperature dependences has attracted maximum attention because of its superior characteristics, which make it suitable for practical applications. One of these characteristics is tunable heat flux, controlled by the thickness of the composites. In addition, the geometry of the bulk thermal rectifier is not subject to any significant physical constraints, so it can be easily incorporated into a wide range of mechanical components.

The principle of the bulk thermal rectifier, which is a composite consisting of two solid materials joined together, each possessing thermal conductivities (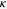) with different temperature dependences, was theoretically proposed by two different groups in 2006 [11, 12]. These theoretical predictions were subsequently validated by experiments [13–15]. Despite the experimental confirmation of bulk thermal rectification, several problems prevent their use in practical applications. One of the most serious problems is the very low working temperature. Thermal rectification in a bulk material was first observed at low temperatures below 150 K [13–15] because these composites made use of the significant temperature dependence of lattice thermal conductivity, typically observed in crystalline materials below 100 K. Unfortunately, increasing the temperature range over which this large variation in lattice thermal conductivity occurs is difficult, especially up to room temperature (300 K). Another problem is the small magnitude of the thermal rectification ratio (TRR = 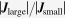) observed for the bulk thermal rectifiers; the largest value observed is less than 1.45 [13–15], which would not be suitable for applications. To make a practical bulk thermal rectifier, we need to greatly increase both the working temperature and the magnitude of the TRR.

We considered that the serious problems could be removed if we could employ an Al-based icosahedral quasicrystal (IQC) as a main component of the bulk thermal rectifier because it is characterized by drastically increasing thermal conductivity with an increasing temperature above 300 K [16, 17]. Indeed, by making composites consisting mainly of IQC, we succeeded in developing new thermal rectifiers that work at high temperatures above 300 K. In this paper, we shall report the performance of our newly developed thermal rectifier, together with the detailed information about the unusual thermal conductivity of Al-based IQC.

## 2. Unusual electron thermal conductivity of icosahedral quasicrystals

The thermal conductivity of Al-based IQC is characterized by its small magnitude at low temperatures below 300 K and the drastic increase with increasing temperatures (at high temperatures above 300 K). The former is caused by the small magnitude of both the lattice thermal conductivity and electron thermal conductivity. The small lattice thermal conductivity in IQCs is realized due to the quasiperiodicity and its corresponding phonon dispersions in which optical phonon branches exist in the low energy range [18], causing a significant reduction of group velocity and the enhancement of the Umklapp process of phonon scattering. The quasiperiodicity also contributes to the small electron thermal conductivity by enhancing the scattering probability of electrons into the strongest scattering limit known as the Mott–Ioffe–Regel limit [19]. The very small electronic density of states at the Fermi level [20–22] limits the number of conducting electrons and further reduces the magnitude of electron thermal conductivity [23, 24].

Despite that, the lattice thermal conductivity is kept small at high temperatures, and the magnitude of electron thermal conductivity of IQCs drastically increases with the increasing temperature. This unusual electron thermal conductivity is caused by the narrow, deep pseudogap at the Fermi energy.

The electron thermal conductivity is formulated in the context of the linear response theory [25].

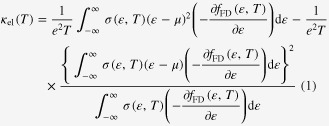
Here, 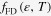, 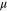 and *e* represent the Fermi–Dirac distribution function, the chemical potential and the unit charge of electron, respectively. The function 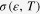 is known as the ‘spectral conductivity’ that represents the contribution of electronic states existing at 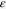 to the electrical conductivity. If we use the relaxation time approximation with the isotropic electronic structure, the spectral conductivity is expressed by the following equation

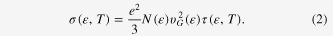
Here, 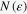, 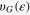, and 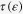 are the electronic density of states, the group velocity and the relaxation time, respectively. These three values generally vary with energy and temperature, and the resulting 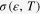 also shows significant energy dependence and strong temperature dependence.

**Figure 1.**
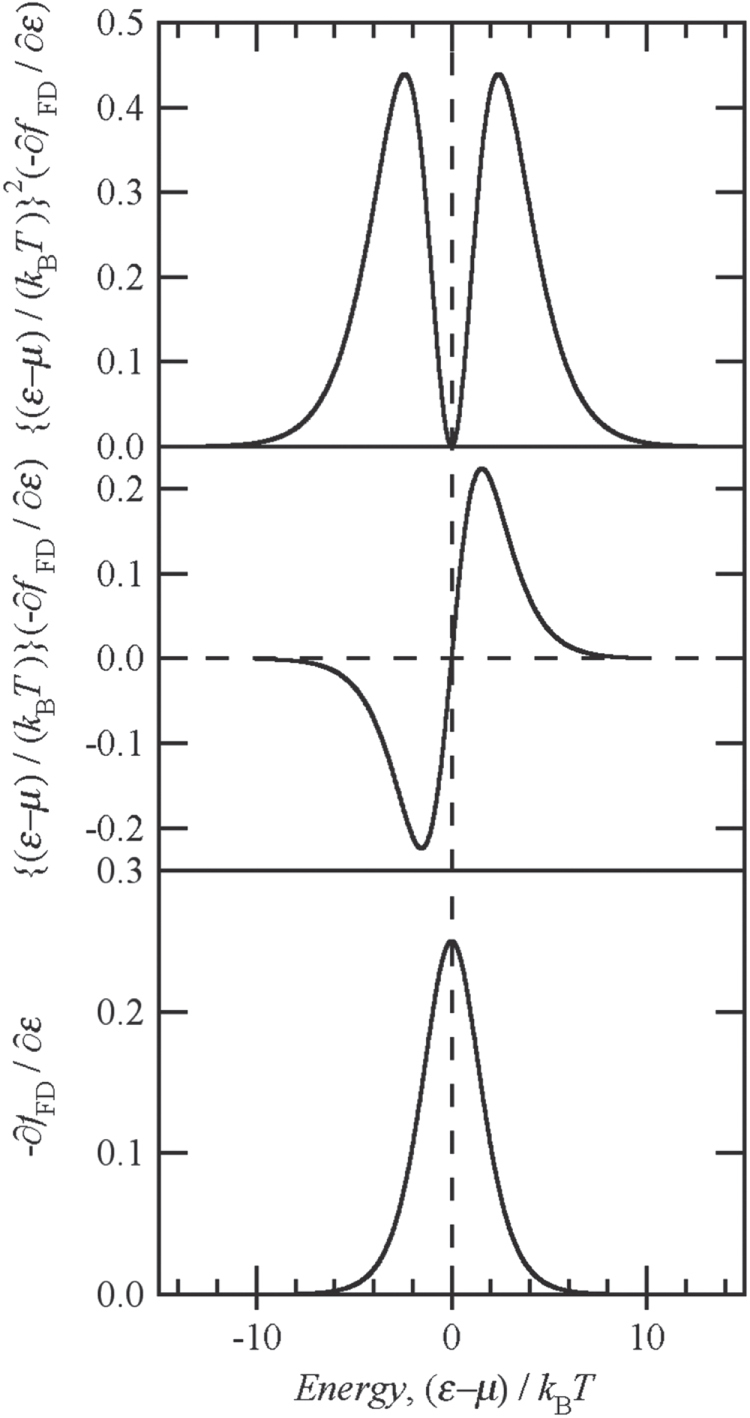
Three-window-functions limiting the energy range of electrons that contribute electron transport properties. The electron thermal conductivity is mainly determined by the function at the top panel.

When the Seebeck coefficient is smaller than 100 *μ*V K^−1^ [23, 24], the second term of equation (1) is small and can be safely ignored. In such a case, the behavior of electron thermal conductivity is accounted for solely with the first term of equation (1). The integrant in the first term of equation (1) contains 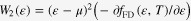, which behaves as a window function and determines the energy range of electrons that contribute to the thermal conductivity, as shown in figure 1. Obviously, it has two peaks below and above the chemical potential *μ* at around 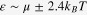.

In the case of IQCs, 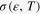 has almost temperature independence and becomes directly proportional to 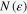 because of the quasiperiodicity and consequently introduces a strong scattering limit. Let us now assume that 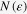 has a deep, narrow pseudogap of a few hundred meV in width, and the Fermi energy is located at the energy where 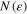 possesses the smallest magnitude in the pseudogap. At a low temperature, 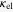 should be kept very small because of the very small magnitude of 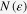 at 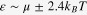, while it drastically increases with the increasing temperature because the electronic density of states drastically increases with being apart from the Fermi energy; consequently, *N*(*∊*) at 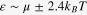 drastically increases with the increasing temperature. This is the reason why we observe a large evolution of electron thermal conductivity, and this mechanism was quantitatively investigated using the Al-Re-Si and Al-Mn-Si 1/1-cubic approximants [23, 24].

Dolinšek *et al* [26] reported that 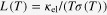 of Al-Cu-Fe IQC becomes almost constant above 300 K in their calculation using equation (1). Their result is certainly inconsistent with our interpretation of the unusual evolution of the thermal conductivity of IQCs. We should comment on this inconsistency and mention that the model of spectral conductivity used by Dolinsek *et al* was too simplified for estimating thermal conductivity at high temperatures above 300 K. Their spectral conductivity consisted of two Lorentzian functions in the same manner as that reported by Landauro and Solbrig [27], and the detailed shape was determined so as to reproduce the measured transport properties below 300 K using the equations deduced from the linear response theory. Their spectral conductivity should be highly reliable in the narrow energy range near the Fermi energy because of the function fitting on experimental data. However, it should not be reliable at the energy range apart from the Fermi energy where high temperature properties are determined because the transport properties below 300 K do not contain the information about the spectral conductivity at that energy range. This should be the reason why Dolinšek *et al* [26] reported behavior of electron thermal conductivity that is certainly inconsistent with our interpretation.

The reliability of our argument on the unusual increase of electron thermal conductivity can be confirmed in the experimental facts: (1) the magnitude of the Seebeck coefficient is closely related with the evolution of thermal conductivity at high temperatures [22], (2) the electron thermal conductivity of 1/1-cubic approximants shows almost the same behavior of that calculated from the electronic density of states determined theoretically and experimentally [23, 24] and (3) the behavior of thermal conductivity sensitively varies with carrier concentration [23, 24, 28]. These facts lend great support to our scenario of an unusual increase of electron thermal conductivity at high temperatures.

## 3. Thermal conductivity of Al-Cu-Fe icosahedral quasicrystals

As a result of the analyses on the electronic structure and its relation to the unusual behavior of electron thermal conductivity [23, 24], we realized that the Al-based IQC and their corresponding approximants show a significant increase of thermal conductivity with the temperature, provided that those IQCs and their approximants contain 3d transition metal elements as one of the main constituent elements, rather than 4d or 5d transition metal elements. This tendency is caused by the narrower width of the pseudogap in IQC, which contains 3d transition metal elements. The narrower width of the pseudogap for the IQC and their approximants containing 3d transition metal elements is also understood from the behavior of the Seebeck coefficient, which increases with the increasing temperature and starts to decrease after becoming maximal at the *T*_peak_. The peak temperature *T*_peak_ roughly represents the width of the pseudogap, and the IQCs and their approximants containing 3d elements possess lower *T*_peak_ than that those containing 4d and/or 5d elements [29, 30]. These considerations, together with the very small electronic density of states at the Fermi energy reported for Al-Cu-Fe IQC [20], prompted us to employ the Al-Cu-Fe IQC for the most appropriate material possessing a drastic increase of electron thermal conductivity with increasing temperatures.

**Figure 2.**
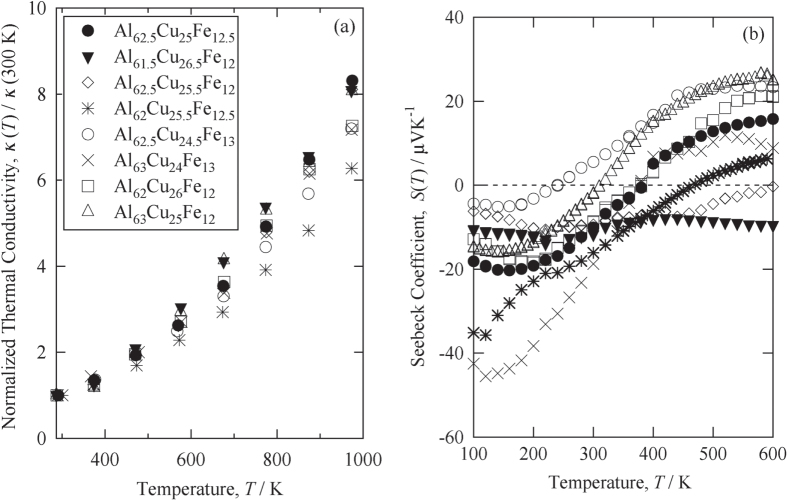
(a) Normalized thermal conductivity and (b) Seebeck coefficient of Al-Cu-Fe icosahedral quasicrystals. At the composition where the temperature dependence of the Seebeck coefficient becomes less significant, the thermal conductivity possesses the most significant increase with the increasing temperature.

Figure 2(a) shows the thermal conductivity of Al-Cu-Fe IQC. The mother ingots of Al-Cu-Fe IQC were prepared by induction melting under a pressurized argon atmosphere. The ingots were crushed into powders and sintered using a pulse-current sintering technique for making dense samples free of voids and cracks [28, 31]. Since electron thermal conductivity of IQC sensitively varies with the carrier concentration, we prepared several different samples possessing different carrier concentrations. We also measured the Seebeck coefficient of the samples (see figure 2(b)) because the magnitude of the Seebeck coefficient is supposed to be small when the electron thermal conductivity shows a drastic increase at high temperatures. The magnitude of the Seebeck coefficient becomes small at Al_61.5_Cu_26.5_Fe_12_ where the ratio of thermal conductivity at 1000 K to that at 300 K, 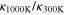 possesses the largest value of 8.9 [28]. Therefore, we decided to employ this Al_61.5_Cu_26.5_Fe_12_ IQC as one of the main components of the thermal rectifier.

## 4. Materials possessing thermal conductivity that decreases with increasing temperatures

**Figure 3.**
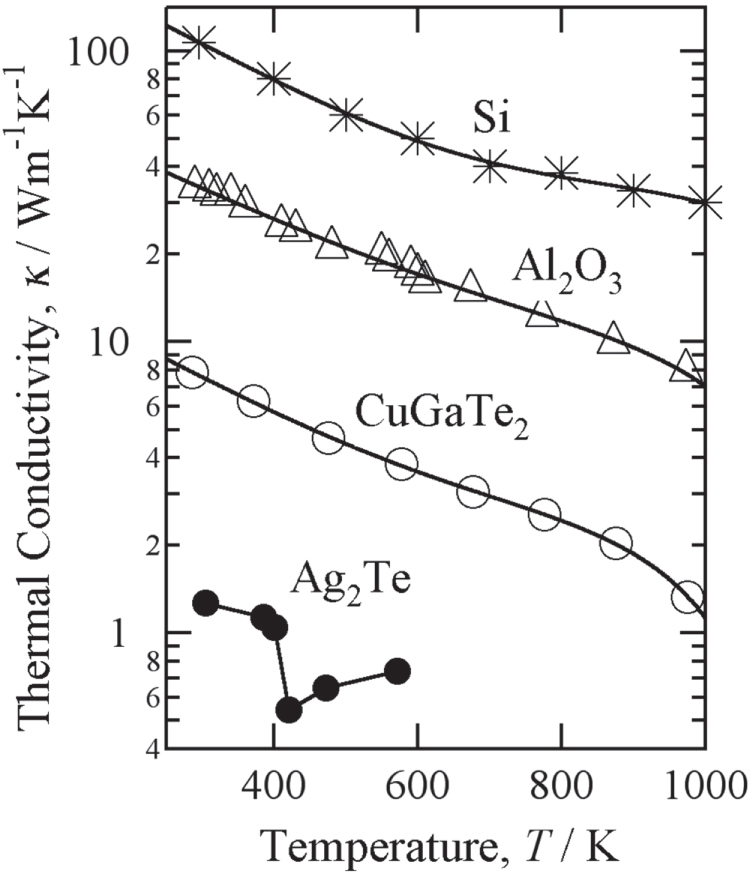
Thermal conductivity of Si, Al_2_O_3_, CuGeTe_2_ and Ag_2_Te. All of the samples possess a decreasing thermal conductivity with the increasing temperature.

We selected Si, Al_2_O_3_, CuGeTe_2_ and Ag_2_Te as the materials possessing thermal conductivity that decrease with increasing temperatures. Figure 3 shows the thermal conductivity of these materials plotted as a function of temperature.

The downward trend in thermal conductivity with increasing temperatures observed for Al_2_O_3_ and Si is easily understood as a consequence of the lattice thermal conductivity of an insulator possessing a high Debye temperature (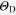). These values have been reported as 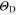 = 1000–1100 K for Al_2_O_3_ [32] and 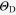 = 600–650 K for Si [33]. In such materials, lattice thermal conductivity moderately decreases with the temperature as a result of the intensified Umklapp process of phonon-phonon scattering.

The temperature-dependent behavior of *κ* in CuGaTe_2_, on the other hand, is difficult to interpret. Its Debye temperature was reported to be very low: 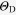 = 226 K [34], and this low 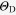 indicates that lattice vibrations at high temperatures (above 200 K) should not be considered as ‘conducting wave packets’ but rather as ‘intensely exited, localized oscillators.’ In materials such as these, the lattice thermal conductivity should have no temperature dependence at high temperatures (*T* > 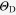), but this consideration is not the case with the experimentally observed variation of *κ* of CuGaTe_2_. Although the mechanism that produces large reductions in *κ* with the increasing temperature is not yet well understood, we are justified in employing CuGaTe_2_ as one of the components of our rectifier partly because its thermal conductivity varies with temperature more significantly than that of Al_2_O_3_ or Si and partly because its *κ* is very small to be comparable with that of the IQC.

The thermal conductivity of Ag_2_Te shows a big reduction at 420 K with increasing temperatures [35]. At temperatures above 420 K, silver ions start to wander in the samples [36]. The mobile silver ions presumably prevent the propagation of the wave packet or directly prohibit the existence of well-defined wave packets; therefore, the lattice thermal conductivity shows very small values.

## 5. Calculation of thermal rectification ratio TRR


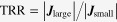
 is determined not only by the temperature dependence of the thermal conductivity of two constituent materials but also by their length ratio *x* = 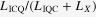 (*X* = Si, Al_2_O_3_, CuGeTe_2_, or Ag_2_Te). Before preparing the composite samples, we estimated the optimal length ratio *x*_opt_ for the maximum TRR obtainable for the given set of materials. The calculation method was reported previously [31].

**Figure 4.**
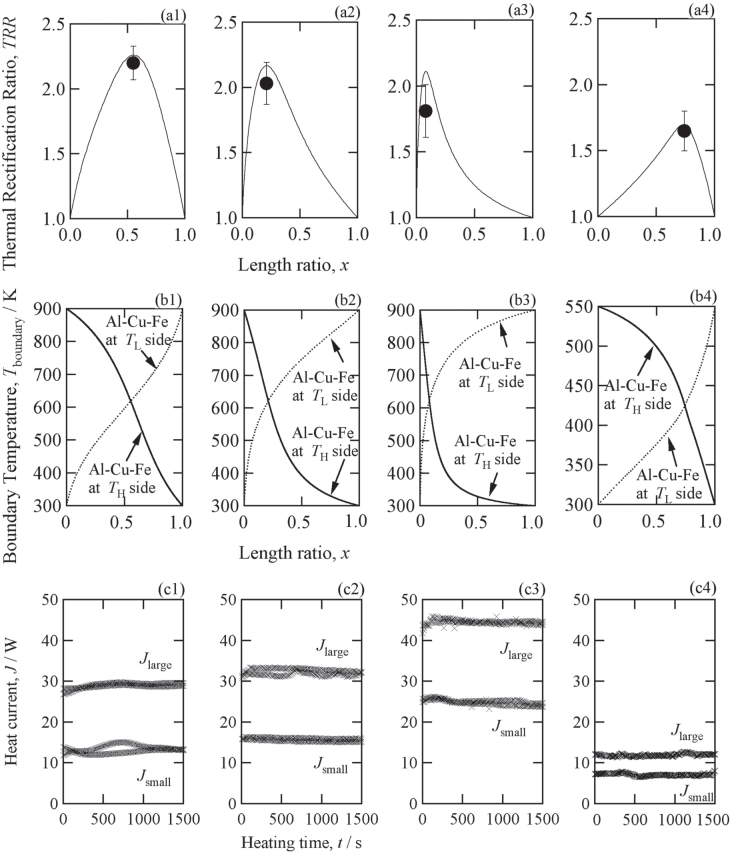
(a) Thermal rectification ratio, (b) boundary temperature and (c) measured heat flux of the (1) IQC/Si, (2) IQC/Al_2_O_3_, (3) IQC/ CuGaTe_2_ and (4) IQC/Ag_2_Te composites.

The *x* dependence of TRR was calculated for the composite thermal rectifiers consisting of (a) Al_61.5_Cu_26.5_Fe_12_ IQC/Si, (b) Al_61.5_Cu_26.5_Fe_12_ IQC/Al_2_O_3_, (c) Al_61.5_Cu_26.5_Fe_12_ IQC/CuGeTe_2_ and (d) Al_61.5_Cu_26.5_Fe_12_ IQC/Ag_2_Te by assuming that the composites are placed between two heart reservoirs kept at (*T*_H_, *T*_L_) = (900 K, 300 K) for (a)–(c), and (*T*_H_, *T*_L_) = (543 K, 300 K) for (d). The resulting TRR is plotted as a function of *x* in figures 4(a1)–(a4).

The calculations predict that very large values of TRR exceeding 2.0 can be obtained in three composites: IQC/Si, IQC/Al_2_O_3_, and IQC/CuGaTe_2_, placed between two heat reservoirs kept at 900 K and 300 K. Each composite has its own optimum value of *x* (*x*_opt_) in which the largest value of TRR_calc_ is attained. The *x*_opt_ values were 0.08, 0.21 and 0.55 for IQC/Si, IQC/Al_2_O_3_ and IQC/CuGaTe_2_, respectively.

It is also worthwhile to mention that the large value of TRR exceeding 1.75 is obtainable even at the small temperature difference from 543 K to 300 K in the composite of ICQ/Ag_2_Te when the length ratio is *x* = 0.65. Although the predicted value of TRR_calc_ = 1.75 is slightly smaller than those of the other three composites, the much smaller temperature difference of *ΔT* = 243 K than *ΔT* = 600 K of the other composites could have advantages in practical applications.

The conditions that determine *x*_opt_ are clearly understood from the *x*-dependence of the interface temperature, as shown in figures 4(b1)-(b4).

At *x*_opt,_ the interface temperature (*T*_boundary_) possesses the same value for two different sample configurations: one configuration with the IQC at the high-temperature side and the other with the IQC at the low-temperature side. This result indicates that the ratio of the thermal resistance of the IQC at the high temperature side to that of the one of the other materials at the low temperature side becomes equal to the ratio of thermal resistance for the one of the other materials at the high temperature side to that of the IQC at the low temperature side.

In addition, *x*_opt_ requires a longer length of the material with the larger thermal conductivity, and vice versa. We obtained the smallest value of *x*_opt_ in the IQC/Si composites, because Si possesses a much larger *κ* than the Al_61.5_Cu_26.5_Fe_12_ IQC. *x*_opt_ is in the vicinity of 0.5 for the IQC/CuGaTe_2_ and IQC/Ag_2_Te composites because of the comparable *κ*-values of the two components. The calculated *x*_opt_, the boundary temperature at *x*_opt_ and the estimated maximum value of TRR_calc_ are listed in table 1.

**Table 1.** Thermal rectification ratio of the present composites.

Material A	Material B	*T*_H_/K	*T*_L_/K	TRR_exp_	TRR_calc_	TRR_exp_/TRR_calc_
Al_61.5_Cu_26.5_Fe_12_ IQC	Si	900	∼300	1.81 ± 0.16	2.10	0.86
Al_61.5_Cu_26.5_Fe_12_ IQC	Al_2_O_3_	900	∼300	2.01 ± 0.13	2.17	0.92
Al_61.5_Cu_26.5_Fe_12_ IQC	CuGaTe_2_	900	∼300	2.20 ± 0.13	2.26	0.97
Al_61.5_Cu_26.5_Fe_12_ IQC	Ag_2_Te	550	∼300	1.65 ± 0.16	1.75	0.96

## 6. Heat flux measurement for determining the TRR

To experimentally confirm the very large values of the TRR predicted from our calculations, we directly measured the heat flux in the IQC/Si, IQC/Al_2_O_3_, and IQC/CuGaTe_2_ composites placed between two heat reservoirs kept at *T*_L_ ≈ 300 K and *T*_H_ = 900 K in vacuum and that in the IQC/Ag_2_Te placed at *T*_L_ ≈ 300 K and *T*_H_ = 550 K.

The samples have cylindrical shapes 20 mm in height and 10 mm in diameter, and the length ratios *x* were fixed at *x*_opt_ for all of the composites. The heat flux *J* in the composite was estimated using a simple apparatus comprised of a heater, one of the composites, a copper block and a water-cooled block placed in a hand-press. The entire measurement system was sealed in a chamber, and the measurements were conducted under vacuum better than 10 Pa [28, 31]. To obtain good thermal contacts, both sides of the cylindrical ingots were carefully polished to a flat, mirror-like finish, and the cylindrical ingots were joined using a small amount of silver paste. We also used a thin carbon sheet (0.5 mm in thickness) at the interface between the sample and heater. A small amount of thermal grease was employed at the interfaces between the sample/copper block and the copper block/water-cooled block.

The measured heat fluxes are plotted in figures 4(c1)–(c4) as a function of heating time. Large differences in heat flux were observed between the measurements made in the two opposite directions, and the heat flux is always larger when the IQC is located at the high-temperature side. The experimentally observed TRR (TRR_exp_) values for the IQC/Si, IQC/Al_2_O_3_, IQC/CuGaTe_2_ and IQC/Ag_2_Te composites were 1.81 ± 0.08, 2.03 ± 0.16, 2.20 ± 0.13 and 1.65 ± 0.20, respectively. These values are much larger than those of any other bulk thermal rectifiers previously reported [13–15, 28, 31, 37].

## 7. Discussions

The TRR_exp_ values were nearly consistent with the TRR_calc_ values for the IQC/CuGaTe_2_ and IQC/Ag_2_Te devices, whereas the other two rectifiers possessed TRR_exp_ values that were slightly smaller than TRR_calc_. This fact is clearly confirmed by superimposing the TRR_exp_ data on the TRR_calc_ data in figure 4(a). Additionally, by calculating the ratio of TRR_exp_ to TRR_calc_, we discovered that this ratio is closely related to the averaged thermal conductivities of component materials, estimated from the temperature distribution in the composites and the temperature dependence of *κ* for each material. The values of TRR_exp_/TRR_calc_ were 0.97, 0.96, 0.92 and 0.86 for IQC/CuGaTe_2_, IQC/Ag_2_Te, IQC/Al_2_O_3_ and IQC/Si, respectively. Their averaged *κ* for the two different directions of heat flow were calculated to be (2.78 W m^–1^ K^–1^, 6.08 W m^–1^ K^–1^), (1.55 W m^–1^ K^–1^, 2.63 W m^–1^ K^–1^), (7.48 W m^–1^ K^–1^, 15.97 W m^–1^ K^–1^) and (19.53 W m^–1^ K^–1^, 40.93 W m^–1^ K^–1^), respectively. Very small values of TRR_exp_/TRR_calc_ were obtained for the IQC/Si composite, which possesses the largest values of averaged *κ*, whereas TRR_exp_/TRR_calc_ reached nearly equal to unity in the IQC/CuGaTe_2_ and IQC/Ag_2_Te composites, which possess the very small values of averaged *κ*.

We firstly considered the effect of radiation emitted from the sidewall of the samples. The amount of radiation loss is not negligibly small but occasionally reaches a seriously large value. It is strongly affected by the dimension and thermal conductivity of the samples and becomes large for long, narrow samples of lower thermal conductivity. In the present samples with cylindrical shapes, we confirmed that the radiation loss was less than 10%, and the variation of TRR due to the radiation was less than a few %. Therefore, we safely ignored the effect of radiation loss in our samples.

**Figure 5.**
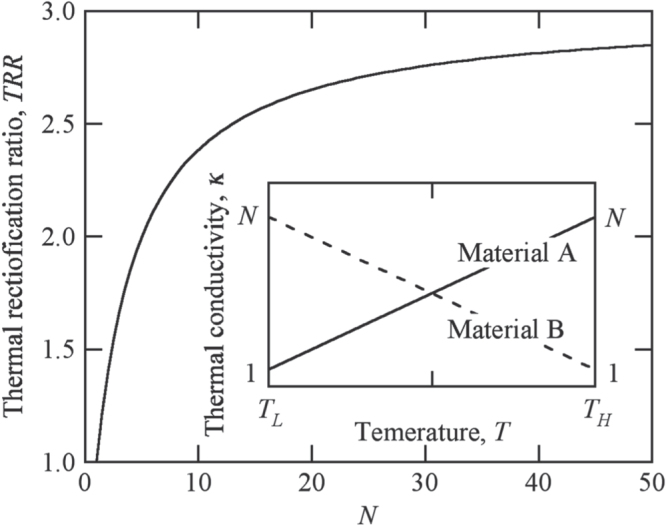
Thermal rectification ratio obtainable for composites consisting of material A and material B, which possess thermal conductivity that linearly varies with temperature.

We eventually realized that the departure of TRR_exp_/TRR_calc_ from unity would be related to the contact resistance for heat flow at the interfaces between the heat reservoirs and the devices because it is capable of significantly reducing the highest temperature or significantly increasing the lowest temperature of the composite. The thermal conductivity of the Al_61.5_Cu_26.5_Fe_12_ IQC and the CuGaTe_2_ was small enough so that the effect of the contact resistance could be safely ignored; therefore, TRR_exp_/TRR_calc_ was nearly equal to unity for the IQC/CuGaTe_2_ composite. In the case of the IQC/Si composite, on the other hand, the thermal conductivity of Si is so large that the temperature distribution in the samples is greatly affected under the presence of contact resistance. The highest temperature of the composite would be reduced significantly when Si is located at the hot side, while the lowest temperature would be increased at the opposite configuration. The temperature difference between the two edges of the composite is certainly reduced, and the value of the TRR is also reduced.

The contact resistance between two component materials, on the other hand, does not seriously affect the value of the TRR because the value is dominated by the thermal conductivity near the highest and lowest temperatures, whereas the interface of two component materials always stays in the middle temperature range.

If our consideration is correct, we still have a chance to observe a large TRR exceeding 2.0 even for the IQC/Si composite by tuning the heater power using the temperature exactly at the interface rather than that in the heater block. This requires the modified experimental setup; we are now in progress on it, and the results will be reported elsewhere in the near future.

Before the closing discussion, it would be worthwhile to mention the further increase of the TRR. Let us assume a composite consisting of two materials, one of which possesses thermal conductivity that linearly increases with temperature, and the other linearly decreases. For the sake of easy calculation, the increasing ratio and decreasing ratio of thermal conductivity in two materials were assumed to be the same as 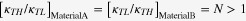. The value of TRR_model_ was calculated as a function of *N* under this condition, and the resulting values were plotted in figure 5.

Obviously, the TRR increases with increasing *N* and gradually approaches the maximal value 3. The Al-Cu-Fe IQC possesses *N* = 7.0 under the condition of *T*_H_ = 900 K and *T*_L_ = 300 K, and the TRR ∼ 2.2 observed for the composite consisting mainly of this IQC is very close to the TRR_model_ at *N* = 7.0. These facts indicate that our samples have already stayed in very good condition for the thermal rectifier and that it would not be very easy to obtain much larger TRR values exceeding 3.0.

Nevertheless, if we employed materials possessing thermal conductivity that increases with temperature much more drastically than those with linear dependence, the maximum value should be increased to a value larger than 3.0. The thermal conductivity of IQCs possesses such a behavior; therefore, we keep studying toward the goal of developing a thermal rectifier possessing a TRR exceeding 3.0 using IQCs. The result will be reported elsewhere in the near future.

## 8. Conclusion

In this study, we developed a new thermal rectifier working at high temperatures above 300 K and possessing a large TRR exceeding 1.65 using Al_61.5_Cu_26.5_Fe_12_ IQC together with one of the following materials: Si, Al_2_O_3_, CuGeTe_2_ or Ag_2_Te. The values of TRR obtained in this study were definitely the largest among those ever reported; therefore, some of these composites could be used in practical applications, leading to the efficient use of energy.
